# Telemedicine Applications in Pediatric Retinal Disease

**DOI:** 10.3390/jcm6040036

**Published:** 2017-03-23

**Authors:** Akhilesh S. Pathipati, Darius M. Moshfeghi

**Affiliations:** 1 Stanford University School of Medicine, Stanford, CA 94305, USA; apathip@stanford.edu; 2 Department of Ophthalmology, Byers Eye Institute, Horngren Family Vitreoretinal Center, Stanford University School of Medicine, Palo Alto, CA 94303, USA

**Keywords:** teleophthalmology, retina, pediatrics, retinopathy of prematurity, telemedicine screening

## Abstract

Teleophthalmology is a developing field that presents diverse opportunities. One of its most successful applications to date has been in pediatric retinal disease, particularly in screening for retinopathy of prematurity (ROP). Many studies have shown that using telemedicine for ROP screening allows a remote ophthalmologist to identify abnormal findings and implement early interventions. Here, we review the literature on uses of telemedicine in pediatric retinal disease and consider future applications.

## 1. Introduction

Telemedicine broadly refers to the use of communications technology to assist in the diagnosis and management of disease [1]. Recent advances in ophthalmic imaging and mobile technology have enabled applications in ophthalmology. 

Efforts to date have focused on screening and monitoring of disease [2]. For instance, telemedicine-based screening of diabetic retinopathy (DR) has been shown to increase the proportion of patients receiving an annual exam, identify diabetic changes to the retina, and decrease vision loss from DR at a population level [3,4,5]. Several studies have evaluated telemedicine for glaucoma and age-related macular degeneration as well [6,7,8,9]. Results have been mixed but promising for both conditions.

While these findings are encouraging, teleophthalmology has had limited use in adult populations. Further research is needed regarding its practicality, reliability, and cost-effectiveness [10]. In this context of limited information, patients, providers, and payers have struggled to establish a viable financial model for services. As a result, services remain poorly reimbursed and are not widely available outside of research settings [11].

By contrast, teleophthalmology has had considerable success in the pediatric population, particularly for retinal disease in newborns. It has been widely validated for screening of retinopathy of prematurity (ROP) [12,13] and has more recently been applied as a screening tool for other ocular pathology present at birth [14,15]. 

Pediatric retinal disease is particularly well suited to telemedicine because (1) the existing health care delivery system struggles to screen for these diseases [16,17]; (2) digital imaging tools allow us to address that need [18,19]; and (3) once a condition is identified, there are often therapeutic interventions available that benefit the patient [20,21]. It therefore avoids the pitfalls facing telemedicine programs that are conceptually appealing but do not have clear targets and ultimately do not improve health. This review will summarize the literature and consider future applications of telemedicine in pediatric retinal disease.

## 2. Background on Retinopathy of Prematurity

Retinopathy of prematurity refers to a condition of immature retinal blood supply in premature and low-birth-weight infants. When an infant is born before retinal vasculature has adequately developed (typically before 30–32 weeks of gestation), areas of the peripheral retina may become ischemic. This ischemic retina secretes vascular endothelial growth factors (VEGF) that can lead to disordered neovascularization, retinal detachment, and blindness. Although rare, ROP is a leading cause of blindness in children and is believed to affect a majority of preterm infants born below 1500 g [20].

ROP is further categorized by geographic zones of the retina that are involved (Zones 1–3), staging of the junction between vascular and avascular retina (Stages 1–5), and the presence of plus disease. Plus disease refers to a set of characteristic complications relating to vascular dilation and tortuosity that can be present in any stage [22,23].

Infants are deemed candidates for treatment based on zone, stage, and the presence of plus disease. Surgical treatment is recommended for Zone I (any stage with plus disease), Zone I (Stage 3 without plus disease), or Zone II (Stage 2 or 3 with plus disease) [24]. 

Treatment options include ablative therapy, cryotherapy, laser surgery, and intravitreal injections [20,21]. Randomized trials have shown that early treatment significantly improves outcomes and effectively preserves vision [20,21]. Screening therefore provides a substantial clinical impact.

## 3. Telemedicine for Retinopathy of Prematurity

Current guidelines recommend bedside screening of at-risk infants in the neonatal intensive care unit (NICU) by binocular indirect ophthalmoscopy (BIO); in fact, ROP screening is required for NICUs to maintain accreditation in the United States (US) [24]. Meeting this standard is challenging for various reasons. First, there is an increasing number of preterm infants who are born and who live as neonatal care becomes more sophisticated. At the same time, there is a decreasing number of ophthalmologists willing to perform ROP exams due to medicolegal liability and the logistical hurdles of traveling to NICUs and coordinating with staff for eye exams [16,17]. As a result, many NICUs in the US struggle to provide ROP screening. The challenge is even more pressing abroad.

Telemedicine offers an opportunity to alleviate that burden [25]. Trained NICU technicians can use a wide-angle, fiber optic fundus camera (e.g., RetCam III, Clarity Medical Systems, Pleasanton, CA, USA) to obtain retinal images and send them to a specialist via remote digital fundus imaging (RDFI). The specialist can then screen images for ROP.

A host of studies have evaluated RDFI dating back to the early 2000s [13]. Existing research indicates that telemedicine is a reliable, cost-effective method for ROP screening that is comparable to bedside BIO [2,26,27,28]. 

The photographic screening for retinopathy of prematurity study (PHOTO-ROP) was one of the early studies to evaluate telemedicine for ROP [26]. It enrolled 51 infants in a prospective, multicenter trial in 2001 and 2002. The study found that RDFI detected “clinically significant” ROP with 92% sensitivity and 37% specificity compared to BIO. For patients with ROP that met criteria for early treatment of ROP, RDFI demonstrated 92% sensitivity and 67% specificity.

Quinn et al. evaluated 1257 infants for “referral warranted” ROP (RW-ROP) using RDFI [27]. Digital image grading by non-physician readers identified RW-ROP with 90% sensitivity and 87% specificity compared to BIO by an ophthalmologist.

In some respects, wide-angle photography is even superior to BIO. Richter et al. found that ROP screening via telemedicine required less time than BIO, thereby increasing the efficiency of the examining ophthalmologist [29], while Myung et al. noted that digital image capture allowed for analysis of the progression of ROP over time [30].

In light of these findings, the 2013 screening guidelines acknowledge the value of telemedicine in ROP screening [24] and allow for telemedicine as an alternative means of ROP screening. A subsequent Joint Technical Report issued by the American Academy of Pediatrics in 2015 found that “There is level I evidence from at least 5 studies demonstrating that digital retinal photography has high accuracy for the detection of clinically significant ROP” [12]. 

Of note, RDFI does not capture all ROP. The accuracy of wide-angle cameras is not as well established for mild ROP that is primarily present in Zone 3 [12]. However, this type would not require treatment. RDFI is capable of identifying clinically actionable ROP with a high degree of accuracy—the relevant goal for a screening program.

Multiple studies have reported on “real world” programs that employ telemedicine for ROP screening. The Stanford University Network for Diagnosis of Retinopathy of Prematurity (SUNDROP) is an ongoing telemedicine screening program (founded by author DMM). Nurses in underserved NICUs capture retinal images that are reviewed by a retinal specialist at a remote quarternary care center [18]. Now, with more than 11 years of data and over 1000 infants screened, the remote interpretation of RetCam images has had 100% sensitivity and 99.8% specificity compared to BIO for treatment-warranted ROP [18]. In other words, no blinding disease has been missed by telemedicine screening of ROP.

Similarly, Lorenz et al. report on a 6-year, multi-center study in Germany and found that wide-field digital imaging captured all treatment-requiring ROP [31]. The KIDROP program found that a telemedicine screening program effectively identified ROP in rural South India, with 0 infants progressing to unscreened Stage 4 or 5 retinopathy [19]. Finally, the PCA-PERP program in Hungary found that telemedicine screening not only had high diagnostic performance but also produced cost savings [32].

Taken together, these findings demonstrate that telemedicine is an effective modality for ROP screening. While its use should continue to be refined [33], it addresses an important need that the existing ophthalmology infrastructure is not well equipped to handle and does so without sacrificing quality of care.

## 4. Future Applications

Screening for ROP through remote image interpretation is the most well established application of telemedicine for pediatric retinal disease. Additional approaches are now being explored.

### 4.1. Use of Image Analysis Software

Within screening for ROP, there is some excitement about the use of image analysis software to assist in the diagnosis of disease. Identifying plus disease is particularly challenging for clinicians, and a computer software that can trace and quantify vessel characteristics may aid in diagnosis [34]. In one study, a computer program (ROPtool) detected plus disease with 97% sensitivity and 94% specificity, compared to just 72% average sensitivity for human examiners [35].

However, the use of image recognition software faces several challenges. Perhaps most notably, there is poor inter-observer agreement on what constitutes plus disease [36,37]. It is necessary to establish reference standards in order to benchmark a software against those standards.

### 4.2. Universal Screening of Newborns

It goes without saying that vision impairment is common, but it is unknown how much stems from factors present at birth. At present, screening for eye disease typically does not start until several years later, often starting in elementary school. Yet early ocular pathology may be responsible for later ophthalmic disease. 

For instance, amblyopia is the most frequent manifestation of visual impairment in children with estimates of prevalence ranging from 2% to 5% [38]. While the cause is often unknown, it has been hypothesized that untreated perinatal hemorrhage may play a role by influencing the visual axis, eye pressure, or other factors [39]. Detection of early pathology can lead to interventions that dramatically improve vision in the long term.

Given the success of screening for ROP, a logical next step is to incorporate screening for other ocular pathology in newborns. A single-hospital study done in China screened over 8000 otherwise healthy newborns and found ocular pathology was present in 23.2% of cases. Of these, 20.96% were retinal hemorrhages and 2.28% represented other problems (including congenital anomalies, anterior chamber hemorrhage, etc.) [14,40]. 

In order to better understand the effects of ocular abnormalities present at birth, the newborn eye screen testing (NEST) study was initiated at Stanford University. A prospective cohort of newborns at Stanford was enrolled and screened with RetCam III digital photography in 2013–2014. The study will track the development of eye disease over time in this cohort. In the initial enrollment period, 203 subjects were screened with 39 subjects (19%) demonstrating retinal hemorrhages [41]. Along with the NEST study, the Global Universal Eye Screen Testing (GUEST) sub-study will evaluate universal eye screening with images from other parts of the world. 

## 5. Conclusions

Telemedicine has been successfully employed in pediatric retinal disease. It has been most widely used in screening for retinopathy of prematurity but has already shown promise as a universal newborn screening tool. These screening tools benefit from well-defined clinical targets that produce actionable findings. Moving forward, we should continue to prioritize tools that advance health care delivery with targeted, cost-effective approaches.
